# A Case of Suicidal Melancholia, with a General Description of the Disease

**Published:** 1876-08

**Authors:** D. R. Brower

**Affiliations:** Late Superintendent Eastern Lunatic Asylum of Virginia


### REPORT OF A CASE OF SUICIDAL MELANCHOLIA WITH A GENERAL DESCRIPTION OF THE DISEASE,

### AND SOME REMARKS ON ITS MEDICO-LEGAL RELATIONS.

By D. R. BROWER, M. D.,

Late Superintendent Eastern Lunatic Asylum of Virginia.

Mr. A. B. insured his life for the benefit of his wife, in a Connecticut Mutual Life Insurance Company in the year 1866, and committed suicide in the year 1871. In due time his wife presented the necessary evidence of death and demanded the payment of the policy. The Company refused the demand for two reasons, to-wit: first, that the assured died by his own hand, and second, that he had been intemperate during the term of the policy to such a degree as to impair his general health. The policy according to its express provision was rendered null and void by the existence of either of these conditions. The wife brought action in court to recover the sum, and rejoined to the plea of the Company that her husband was insane at the time of his death and not responsible for the suicide, and that he was not intemperate to the degree alleged.

The case was tried before Judge Hopkins of Wisconsin, sitting in the United States District Court of Chicago. The attorney for the plaintiff summoned Drs. Norman Bridge, E. H. Horsey and the writer, all of Chicago, to testify as experts.

The evidence in the case furnished the following history: Mr. A. B. was a carpenter, with very little education; he worked at his trade for a number of years, until impairment of his general health compelled him to abandon it; he then entered upon first the grocery and then the furniture business, but after the great fire in Chicago, came to this city, resumed his trade and continued to work at it until about a week before his death. He had been in the habit of drinking ardent spirits for many years, but not to a sufficient extent to warrant its being called excessive, and for a week previous to his death drank none at all. He was a man of cheerful disposition and social temperament; his family relations had always been pleasant, and no serious calamity or bereavement of any kind had befallen him. About a week before his death he stopped work, said that he was sick, became depressed and solitary in his habits, spent much time in his room alone, had no disposition to indulge in the pastimes of his friends, and insisted that some terrible calamity was about to befall him. He was restless at night; he frequently awoke his bed-fellow to tell him he could not sleep and that he heard strange noises; he complained of pain in his head; he lost his appetite; he at times would complain of feeling very chilly, so much so that he would put his overcoat on and sit close to the stove; he would frequently and in such a manner as to excite the suspicion that something was wrong with him, jump up, leave the room, go out of doors without any apparent object, be gone a short time and return; he would almost every day declare his intention of going to work, but when the time came to start would change his mind, then promise to go the next day, but always with the same result. The morning of the suicide found him unusually cheerful and social, so much so that he played at cards with his comrades, and attended to business, in fact settled up his accounts of partnership work done with them. At noon they separated, his companions went to dinner and he to his bed chamber, and when they next saw him he was lying on his bed dead, with his throat cut, and the pocket knife with which the act was done beside him.

No physician attended him during his sickness and nothing was ascertained concerning his antecedent history nor that of his family.

The experts were called upon to give a general description of suicidal insanity, and then had submitted to them a hypothetical case embracing all the principal points of the evidence above given, and were required to give their opinion as to whether or not this hypothetical case was one of insanity. The experts agreed in opinion that it was.

The defense did not press the plea of inebriety, but they requested that the jury be instructed that not only must the plaintiff prove insanity, but such kind and degree of insanity as would deprive the assured of his capacity of understanding the nature of the act he was about to commit, and the consequences that would result from it ; in other words that the act must have been committed while under the influence of delirium or irresistable insane impulse, in order to avoid its consequences.

The court refused to give the instructions requested by the defense, and read as the rule of law that must govern the case, the opinion rendered by Mr. Justice Hunt, U. S. Supreme Court, in the case of *Life Insurance Co.* v. *Terry*, 15 Wallace, 580, the substance of which is as follows : If the assured being in the possession of his ordinary reasoning faculties, from anger, pride, jealousy, or a desire to escape from the ills of life, intentionally takes his own life, the proviso attaches, and there can be no recovery. If the death is caused by the voluntary act of the assured, he knowing and intending that his death shall be the result of his act, but when his reasoning faculties are so far impaired that he is not able to understand the moral character, the general nature, consequence and effect of the act he is about to commit, or when he is impelled thereto by an insane impulse which he has not the power to resist, such death is not within the contemplation of the parties to the contract, and the insurer is liable.

The jury after deliberating for twenty-four hours were discharged. They had agreed on the question of the man's insanity, but disagreed as to its sufficiency to relieve him from the responsibility of the suicide.

This case of Mr. A. B. is evidently one of suicidal melancholia, and we shall now invite your attention to a succinct description of this form of insanity.

In the first place it is well to state that insanity is a manifestation of cerebral disease, not a disease *per se*, but a symptom of disease ; that it cannot be encompassed within a definition sufficiently comprehensive to include all its manifold manifestations and yet sufficiently circumscribed to exclude those forms of brain disease that are not cases of alienation. Insanity is merely a relative condition, a departure from a condition of sanity, therefore without being well acquainted with the mental manifestation of the brain in a state of health, it is impossible for us to successfully determine and appreciate those manifestations which are the result of disease. It is necessary, in making this comparison, that each mind be compared with itself, inasmuch as no two persons are constituted alike, and hence one may do with impunity many things that if done by another would be regarded as unequivocal evidence of insanity.

In the next place it is well for us to agree upon classification in order to intelligently consider the subject, for alienists differ widely as to the best method of classifying insanity, and have proposed many systems. Without entering upon any discussion of this point, we shall slightly modify an old classification, and divide insanity into three classes, as follows: first, states of mental weakness, including amentia and dementia; second, states of mental exaltation, including mania and monomania; third, states of mental depression, including melancholia. Amentia is unsoundness of mind from arrest of development, either congenital or occurring in childhood, and is divided into idiocy, imbecility, and moral insanity. Dementia is diminished activity, or inertness of the faculties, occurring subsequent to their mature development, is generally a sequence of other forms of insanity, and presents all gradations of mental weakness, from a condition resembling the mild enfeblement of old age to a condition of complete fatuity. Mania and monomania are both characterised by a predominence of exaltation, but differ from each other in the amount of intellectual or emotional impairment.

Melancholia is that form of insanity which is characterised by a predominence of mental depression. This depression, however, is not always present, for it sometimes gives way to an excitement difficult to distinguish from mania, and sometimes to a condition closely approximating the normal mental state of the individual. This form of insanity is frequently only the initiatory stage of mania, monomania, or dementia. The principal cause of melancholia is hereditary transmission, but any cause that will produce debility of the physical structure, or impairment of the moral or intellectual faculties, may result in this form of insanity. Its development is at times so gradual that we fail to notice it until some overt act calls our attention to its existence, and then going back and critically examining the symptoms of our patient we may detect many evidences of derangement that at the time we permitted to pass as too trivial to notice. Its development is at other times very sudden, coming on as the result of some great calamity, or severe nervous or mental shock.

In the beginning of melancholia, the intellectual faculties may be perfectly unimpaired, the only morbid phenomenon being manifest through the affective faculties, the feelings, or emotions of the patient. He may be filled with sorrow, grief, despondency, or serious apprehensions, and fully appreciate his situation, but unable to account for the strange and unindurable misery that overwhelms him. He may attend to his ordinary occupations as usual, and by concealing his emotions from his friends, pass among them as perfectly sane.

As the disease progresses the depression becomes deeper and deeper, the mind dwells more and more constantly on the one idea, the patient takes less and less interest in things around him, forsakes his former associates and dwells in solitude. Still further progress brings intellectual impairment and develops delusions. If the patient is of a religious temperament his delusions will assume the religious type; he will mourn over his lost soul; he will grieve over unpardonable sin; he will imagine himself overwhelmed by horrible crimes he thinks he has committed; he will beg you to save him from impending destruction. If the religious element does not predominate, his delusions may arise from some fancied change in his bodily condition; he may believe himself dangerously ill, suffering from a loathsome and fatal disease, or one of an extremely excruciating character; he may imagine that he has lost the use of his limbs, or that his legs are made of glass, and will allow no one to touch them for fear of breakage; or he may suppose that some horrible animal has taken up its abode in his body. Sometimes this perversion of sensation is the result of impressions propagated by disease, as revealed by *post mortem* examination. For example, a woman who imagined herself pregnant with the devil was found after death to have hydatids of the uterus. At other times they are disconnected with any such impressions. A case is related of a man who was relieved of a snake he imagined in his bowels, by a pretended surgical operation, but afterwards took up the notion that the snake had left its ova behind, and that he would soon be filled with other snakes; but his physician made the ingenious reply that in this he was entirely mistaken, inasmuch as the snake was a male, and thus completely dissipated his delusion.

Notwithstanding the emphatic character of delusions, and the fondness of the patient to dwell upon them, yet the mind may exhibit no impairment of its original vigor, nor any unsoundness whatever on any other topics.

The physical symptoms generally found present in melancholia are, pain, with a sense of weight, throbbing, fullness, giddiness in the head; insomnia, with restlessness; impairment of the functions of the special senses, amounting, in many cases, to hallucination of sight, sound, taste, or smell; anæsthesia, or hyperæsthesia of the skin; impairment of general nutrition and impoverishment of the blood, in consequence of which the body become emaciated, skin pale and sallow, the muscles feeble and relaxed, the strength fails, and in short we may have all the symptoms of anæmia; the digestive system is disordered, the appetite is feeble or capricious, there is sometimes complete anorexia; the respiratory and circulating systems are frequently deranged; the heart's action may be irregular; attacks of palpitation are frequent; the respiration is sometimes very slow and labored, at other times exceedingly hurried; the pulse is feeble and compressible, and the extremities are cold; the menstrual functions are often deranged, and we may have amenorrhœa, menorrhagia, or leucorrhœa, and the temperature will be found below the normal standard.

Suicidal melancholia differs from the melancholia above described only in the suicidal manifestation; the majority of cases of melancholia, particularly those in which hypochondria is present, naturally tend to suicide. It is the result specially to be feared and most carefully to be guarded against in every case.

Suicide is, in the opinion of many people, conclusive evidence of insanity. These people, fortunate in the possession of healthy and well balanced minds, with no neurotic inheritance, cannot in any other way account for so strong and unnatural a phenomena. This opinion, popular as it is, is not sustained by a careful consideration of the subject. There are some, who disbelieving in a future state, will, without hesitation, when met by obstacles in life that seem insurmountable, seek to terminate their existence rather than attempt to encounter them. Many wise men have written in defense of it. The patriotic Roman was taught that it was better to die by his own hand than submit to slavery. The loss of power, wealth, or dearly loved friends, constitute motives sufficient to overpower such as prefer to seek possible good hereafter rather than be compelled to suffer certain misery here; refusing to accept the immortal poet's precept, that it is better

> "To bear those ills we have
> Than fly to others that we know not of."

In all cases of suicide from moral motives alone, that is cases unaccompanied by a pathological condition of the brain, there is always, in our opinion, a motive more or less well defined; it may be of anger, pride, jealousy, or a desire to escape from the ills of life, and when a motive does not exist, it is reasonable to attribute the act to disease. This disease may be unobserved. There are many people engaged in the pursuit of their ordinary occupation, moving along in the even tenor of their ways, totally unsuspected of any mental disturbance, and who might complete their journey of life unsuspected but for some overt act which calls more careful attention to their daily walk and conversation. An interesting case is related as occurring in France, of a man who for over twenty years filled a responsible position under the government with great skill, so much so that he was several times promoted as a reward for his assiduity, his immediate superior in office visited him at regular intervals, and on the occasion of one of the visits, the man shot him dead and immediately killed himself. Their relations had always been agreeable, and the affairs of his office were found to be in perfect condition, but among his papers were found a diary and other writings giving unmistakeable evidence of delusional insanity of many years standing.

The suicidal impulse may be excited in persons predisposed to it by some surrounding circumstances, such as the sight of murderous weapons, standing on the edge of precipices, etc. Most of us have doubtless met with people perfectly sane, whose emotions are so easily aroused that they feel, under certain circumstances, an impulse to self-destruction very difficult to control. We know a legal gentleman distinguished for his great mental ability, who cannot stand at the stern of a vessel in motion and watch the agitation of the water without feeling an almost irresistible impulse to jump over. We have met many persons who cannot ascend a high tower or go to the verge of a precipice and look down, without feeling an intense desire to jump off. In any of these cases let any circumstance hold the intellectual powers of the man at abeyance for a single moment and the suicidal act will be the result.

The suicidal impulse is frequently hereditary. Dr. Rush tells us of a family in which the mother committed suicide, and the two daughters and two sons followed her example. We are told of another family in which the great grandfather, grandfather and father committed suicide.

If the case of Mr. A. B. is tried by the description above given, it will be found to present a sufficient number of symptoms therein described to warrant the diagnosis of suicidal melancholia. The case also directs our attention to the medico-legal relations of this form of insanity.

The courts have established the rule that suicide is a *felo de se* when committed voluntarily by a person who is in the possession of his ordinary reasoning faculties. It then avoids the policy according to the express stipulations of the contract of insurance. The courts very correctly refuse to recognize it as conclusive evidence of insanity, but very greatly err in considering it as the act of a sane and rational mind unless preceded by manifest insanity. The whole subject of the medico-legal relations of the insane has been invested with a dense net-work of unreasonable technicalities and traditional notions. Tests of responsibility have been thrust upon us that fail to recognize the wonderful advance that has been made in the pathology and treatment of insanity, and are made infallible guides in all cases. The insane man is expected to reason correctly upon the questions of right and wrong, to form his conclusions upon the same general principles, and in the same general manner that the sane man does. Equity and common sense have frequently been sacrificed upon the altar of tradition. We are told by jurists that the insane man who commits a crime must be punished to deter other insane men from commiting criminal acts, thereby proclaiming that the insane are capable of the same correct intellectual judgment and moral perception that the sane are. In our humble judgment, on the contrary, the execution of every insane man in the world but one, for homicide, would not in the slightest degree deter the one from committing the same act should he be seized with the homicidal impulse.

The celebrated case of *Borradaile* v. *Hunter*, in many respects similar to the one above described, furnished for many years the rules of law governing all such cases. In this the jury found that the assured voluntarily took his own life, intending so to do, but that at the time of the act he was not capable of judging between right and wrong. Judgment went for the defendant, which was sustained upon appeal to the full bench — thus establishing the remarkable rule that a man who is incapable of judging between right and wrong should be held responsible for criminal acts; a rule, in our opinion, very far removed from equity and common sense. Judge Hopkins showed, in the present case, by giving to the jury the instructions and rules contained in the opinion rendered by Judge Hunt in the case before stated, a much more humane and enlightened appreciation of the peculiarities of the insane mind. This opinion of Mr. Justice Hunt declares, first, that the act of suicide must not only be intentional, but the person committing it must be in the possession of his ordinary reasoning faculties to make it avoid the policy; second, that the death may be the voluntary act of the assured, he knowing and intending that death should be the result of the act, yet he is not responsible if his reasoning faculties are so far impaired that he is not able to understand the moral character, the general nature, consequences and effect of the act; and third, that the man is not responsible if he was impelled to the suicide by an insane impulse.

The liberality of the opinion consists in its first and second declarations, which virtually set aside the judgment in the *Borradaile* v. *Hunter* case, by declaring that suicide may be a voluntary act in all respects and yet carry with it no criminal responsibility. When we consider this opinion in comparison with those previously uttered, we are struck with its great liberality and advancement, and are encouraged with the hope that the day is not far distant when all the courts will abandon the traditions that now invest them when considering the medico-legal relations of the insane, and will weigh each case in the balance of equity by itself, without any arbitary tests of responsibility to complicate the calculation, and will unhesitatingly relieve from criminal responsibility all cases of insanity that eventuate in suicide.

